# Quality of life improvements with setmelanotide treatment in acquired hypothalamic obesity: TRANSCEND trial interview results from US participants

**DOI:** 10.3389/fnbeh.2026.1830070

**Published:** 2026-07-15

**Authors:** Christian L. Roth, Susan A. Phillips, Shana E. McCormack, Katie Larson Ode, Megan M. Kelsey, Jieruo Liu, Claire Ervin, Usha G. Mallya, Lindsey Norcross, Jennifer L. Miller

**Affiliations:** 1 Seattle Children’s Research Institute, Seattle, WA, United States; 2 Department of Pediatrics, Seattle Children’s Hospital, University of Washington, Seattle, WA, United States; 3 Rady Children’s Hospital, University of California, San Diego, San Diego, CA, United States; 4 Division of Endocrinology and Diabetes, Children’s Hospital of Philadelphia, Philadelphia, PA, United States; 5 Department of Pediatrics, Perelman School of Medicine at the University of Pennsylvania, Philadelphia, PA, United States; 6 Department of Pediatrics, University of Iowa–Stead Family Children’s Hospital, Fraternal Order of Eagles Diabetes Research Center, University of Iowa, Iowa City, IA, United States; 7 Department of Pediatrics, University of Colorado School of Medicine and Children’s Hospital Colorado, Aurora, CO, United States; 8 Rhythm Pharmaceuticals, Inc., Boston, MA, United States; 9 RTI Health Solutions, Durham, NC, United States; 10 Pediatric Endocrinology, Department of Pediatrics, College of Medicine, University of Florida, Gainesville, FL, United States

**Keywords:** acquired hypothalamic obesity, hyperphagia, quality of life, setmelanotide, TRANSCEND trial

## Abstract

**Introduction:**

Acquired hypothalamic obesity (HO), caused by hypothalamic injury, is characterized by hyperphagia (i.e., insatiable hunger and abnormal food-seeking behaviors), reduced energy expenditure, and accelerated and sustained weight gain, all of which substantially impact quality of life. In the Phase 3, randomized, double-blind, placebo-controlled TRANSCEND trial (ClinicalTrials.gov identifier: NCT05774756), setmelanotide demonstrated significant improvements compared with placebo in body mass index, hunger, symptoms of hyperphagia, and quality of life in participants with acquired HO. This substudy further characterizes changes in hunger, weight, energy level, and physical activity with setmelanotide and assesses the perceived meaningfulness of such benefits to patients and their caregivers.

**Methods:**

In-depth, 75-min qualitative interviews were conducted from July 2024 to January 2025 with eligible and consented English-speaking TRANSCEND trial participants or their caregivers in the United States. Interviews followed a semistructured interview guide tailored to facilitate discussion about participant experiences or caregiver observations regarding changes in weight, hunger, and energy level before and during trial participation. Interview participants were also asked to describe the perceived meaningfulness of any changes experienced. Interviews were audio recorded and transcribed; thematic analysis was conducted using ATLAS.ti (https://atlasti.com/).

**Results:**

Of the 30 interview participants, 14 were trial participants (4 adolescents and 10 adults) and 16 were caregivers of participants aged < 12 years (*n* = 8) and ≥ 12 years (*n* = 8); 23 received setmelanotide and 7 received placebo. All interview participants reported insatiable hunger and accelerated and sustained weight gain after hypothalamic injury but before starting the trial; 29 of 30 interview participants reported reduced energy level. Consistently, trial participants who received setmelanotide and their caregivers reported beneficial changes in hunger, weight, energy level, and physical activity, and indicated these changes were meaningful, while in placebo-treated participants, no such changes were observed.

**Conclusion:**

These findings support the Phase 3 TRANSCEND efficacy outcomes pertaining to weight, hunger, energy levels, and quality of life and that the impactful changes with setmelanotide in patients with acquired HO and their caregivers extend beyond body mass index and weight reduction.

**Clinical trial registration:**

ClinicalTrials.gov, identifier NCT05774756.

## 1 Introduction

Acquired hypothalamic obesity (HO) is characterized by accelerated and sustained weight gain in the setting of broad impairment following hypothalamic injury often due to suprasellar brain tumors or surgical resection of those tumors, with other causes including traumatic brain injury and meningoencephalitis (Abuzzahab et al., 2025; Argente et al., 2025; Roth and McCormack, 2024). A key driver of accelerated weight gain in acquired HO is reduced stimulation of energy metabolism, contributing to reduced resting energy expenditure, increased fatigue, and lower physical activity, ultimately leading to positive energy balance and weight gain (Abawi et al., 2022; Argente et al., 2025; Hinton et al., 2024; Holmer et al., 2010; Roth and McCormack, 2024).

The melanocortin-4 receptor (MC4R) pathway originating in the hypothalamus is a key regulator of energy balance. By coordinating hunger, satiety, and food intake, it plays a critical role in the homeostatic control of body weight (Heymsfield et al., 2025; Kühnen et al., 2019). Hypothalamic injury causes disruption to the MC4R pathway (i.e., impairment of α–melanocyte-stimulating hormone production), leading to the development of hyperphagia and accelerated, sustained weight gain (Roth et al., 2010). Hyperphagia (defined as a feeling of insatiable hunger and a severe preoccupation with food accompanied by abnormal food-seeking behaviors) is commonly observed in acquired HO (Abuzzahab et al., 2025; Heymsfield et al., 2025). Hyperphagia is considered one of the most distressing symptoms of acquired HO, but its specific impact on both patients and caregivers is not well defined (Abuzzahab et al., 2025). In a survey of 82 caregivers of patients with suspected HO following craniopharyngioma, 72% reported their child also had hyperphagia (Kayadjanian et al., 2023).

In addition to hyperphagia and accelerated weight gain, acquired HO often coincides with reduced energy expenditure and physical activity, fatigue, panhypopituitarism, visual impairment, behavioral disorders, sleep disturbances, and temperature and autonomic dysregulation, which complicate its management (Argente et al., 2025). Acquired HO has a pervasive, negative impact on quality of life (QOL) and a high patient and caregiver burden associated with the multisystem dysfunction, including obesity and hyperphagia (Abuzzahab et al., 2025; Kayadjanian et al., 2023). There are currently no treatments specifically approved for acquired HO. Treatments that have been used in this population (e.g., lifestyle management, earlier-generation glucagon-like peptide 1 [GLP-1] receptor agonists [e.g., exenatide]) do not target the underlying cause of disease in acquired HO and have demonstrated inconsistent efficacy (Dimitri and Roth, 2024; Roth and McCormack, 2024).

Setmelanotide, an MC4R agonist, is approved in the United States, European Union, and United Kingdom for adults and children ≥ 2 years of age for the treatment of obesity associated with impaired MC4R signaling due to proopiomelanocortin deficiency (attributable to biallelic *POMC* variants), proprotein convertase subtilisin/kexin type 1 deficiency (attributable to biallelic *PCSK1* variants), leptin receptor deficiency (attributable to biallelic *LEPR* variants), or Bardet-Biedl syndrome (Clément et al., 2020; Haqq et al., 2022). In addition to significant weight and hunger improvements reported in Phase 3 trials, qualitative reports from patients and caregivers indicate that setmelanotide also contributes to improvements in QOL in those with genetic MC4R pathway–associated obesity and acquired HO (Ervin et al., 2023; Roth et al., 2024b; Wabitsch et al., 2022). In adult patients with acquired HO, setmelanotide demonstrated clinically and statistically significant improvements compared with placebo in body mass index and hunger in the recent Phase 3, randomized, double-blind, placebo-controlled TRANSCEND trial (Miller et al., 2026).

Despite recognition that reduced energy level, increased hunger, and obesity have broad impacts on QOL for patients with acquired HO and their caregivers, (Abuzzahab et al., 2025; Kayadjanian et al., 2023) direct research in this population is limited. To better characterize the treatment benefit associated with setmelanotide in acquired HO, it is necessary to understand the lived experiences of patients with this condition and caregivers. This substudy of the Phase 3 TRANSCEND clinical trial used in-depth qualitative interviews with trial participants and their caregivers to characterize the burden of acquired HO after hypothalamic injury but before initiating treatment in the trial, to evaluate the benefits of setmelanotide treatment, and assess the perceived meaningfulness of the changes following treatment for both patients and their caregivers.

## 2 Materials and methods

Detailed methods of the TRANSCEND Phase 3, double-blind, multicenter, placebo-controlled, international randomized trial (NCT05774756) will be described elsewhere (Miller et al., 2026). Briefly, eligible trial participants (aged ≥ 4 years) had a diagnosis of craniopharyngioma or other brain lesion affecting the hypothalamic region and had undergone surgery, chemotherapy, or radiation therapy involving the hypothalamus or had injury to the hypothalamus for which surgery/radiation was not indicated. Participants were randomized 2:1 to receive setmelanotide or placebo for 52 weeks for a total treatment time of ∼60 weeks (inclusive of a ≤ 8-week dose escalation period). Participants could elect to enroll in the interview portion of the study. Eligible trial participants also had weight gain (either before or after therapy) and body mass index in the 95th percentile or higher (participants aged ≥ 4 to < 18 years) or ≥ 30 kg/m^2^ (participants aged ≥ 18 years). Trial participants were randomized 2:1 to receive setmelanotide or placebo for 52 weeks.

In-depth, 75-min, web-based semistructured qualitative interviews were conducted with a subset of trial participants or caregivers of participants with acquired HO from the TRANSCEND trial (NCT05774756) at participating US-based clinical trial sites. Specifically, study site staff provided the opportunity to all eligible clinical trial participants or their caregivers from participating study sites to consent to voluntarily participate in the qualitative interview substudy. Study staff approached for substudy participation in a consecutive manner until the target of 30 participants consenting to interviews was achieved. Eligibility criteria included the ability to interview in English, age ≥ 12 years, and ability to self-report during a 75-min interview (adolescents: ≥ 12 to < 18 years; adults: ≥ 18 years). For trial participants < 12 years of age or those ≥ 12 years who were unable to self-report, a caregiver for that individual was eligible to participate in this substudy.

### 2.1 Interview methods

All interview participants provided informed consent, assent, or permission (as appropriate based upon their age) for data collection and reporting in adherence to the International Council on Harmonization for Good Clinical Practice. Institutional review board approval was obtained from participating study sites. Interviews were conducted between July 2024 and January 2025 via a web-based platform (i.e., Zoom) within ∼14 days of the end-of-treatment visit. Each interview was conducted by 2 researchers with extensive qualitative research experience and was audio recorded, transcribed, and deidentified to facilitate analysis.

Semistructured interview guides were used to facilitate discussion about trial participant experiences or caregiver observations regarding changes in hunger/hyperphagia, eating behavior, weight, energy level, and physical activity (both after injury to the hypothalamus but before trial entry and after initiation of treatment with the study drug). Complete participant and caregiver interview guides are available as Supplementary material. Interview participants were also asked to describe any impacts on their lives associated with these changes at both time points, and the perceived meaningfulness of any of these changes that occurred after initiating treatment. Interview guides were tailored for trial and caregiver participants to assess the same concepts. Participant or caregiver reported weight loss was converted from pounds to kilograms as applicable.

### 2.2 Analysis

Thematic analysis methods were used to analyze the transcript data; analysis was facilitated by qualitative analysis software [ATLAS-ti 9.0].^1^ Specifically, interview transcripts were coded according to the final codebook; refinement of the final codebook was iterative, with initial codes adapted to incorporate emerging themes based on interview data. During the conduct of the interviews and throughout primary data analysis and reporting, project staff were blinded to treatment assignment. *Post hoc* analysis of key results pertaining to interview participant experiences during the TRANSCEND trial by treatment assignment was conducted following the unblinding of clinical trial data and is included. Analyses were not powered to determine statistical significance, and descriptive statistics were used throughout the substudy.

## 3 Results

### 3.1 Interview participant characteristics

A total of 14 trial participants (4 adolescents, 10 adults) and 16 caregivers completed interviews (Table 1). Among the trial participants interviewed (or whose caregiver was interviewed), 77% (23/30) and 23% (7/30) were randomized to receive setmelanotide and placebo, respectively. The mean age of interviewed adolescent and adult trial participants was 15.0 (range, 13–17) and 28.2 (range, 19–49) years, respectively. Among those whose caregivers were interviewed, the mean age was 7.3 (range, 4–11) and 19.5 (range, 13–24) years for those < 12 years and ≥ 12 years of age, respectively. Baseline demographics of those participating in the substudy were generally similar to the overall study population in terms of sex (∼60% female in the overall study; 70% female in the substudy) and age range (4–66 years in the overall study; 4–49 years in the substudy [Miller et al., 2026]).

**TABLE 1:** Participant characteristics.

Characteristic	Adult trial participants (*n* = 10)	Adolescent trial participants (*n* = 4)	Caregiver participants^a^ of trial participants aged < 12 years (*n* = 8)	Caregiver participants^a^ of trial participants aged ≥ 12 years (*n* = 8)
Trial participant age, mean (SD; range), y	28.2 (8.7; 19–49)	15.0 (1.6; 13–17)	7.3 (2.1; 4–11)	19.5 (4.3; 13–24)
Trial participant sex, nb				
Female	6	3	5	7
Male	4	1	3	1
Trial participant age at time of injury, mean (SD), y^c^	12.5 (7.6; 5–33)	7.5 (2.6; 5–11)	5.0 (2.4; 2–10)	6.6 (2.2; 3–9)
Treatment assignment				
Setmelanotide	8	4	6	5
Placebo	2	0	2	3

SD, standard deviation.

*^a^*Of the 16 caregivers of trial participants, most were mothers (15/16); 1 was the father of a trial participant aged ≥ 12 years.

*^b^*As assigned at birth.

*^c^*Age at time of injury was self-reported. One adult trial participant reported that they experienced injury to the hypothalamus resulting from the growth of a tumor (unrelated to surgery or injury); as such, they could not provide a specific date for the injury and instead provided the date they were diagnosed with acquired hypothalamic obesity. The age at diagnosis was used for the summary statistics.

### 3.2 Patient and caregiver experience following hypothalamic injury and before the TRANSCEND trial

All interview participants reported changes in hunger (i.e., increased hunger intensity and/or frequency) and weight gain following hypothalamic injury, with decreased energy level and physical activity, and mental/emotional, social, and familial burden being reported before starting the trial. Additionally, interview participants commonly reported that they (or their dependents) experienced limited or transient effectiveness with weight management prescription medications, if they had been used.

#### 3.2.1 Impacts and burden associated with hypothalamic injury

All 30 interview participants reported experiencing or observing weight gain following hypothalamic injury and before starting the trial, which was described as rapid and extreme (Table 2 and Box 1). Several caregivers (18.8% [3/16]) reported that their child’s body weight doubled or tripled after hypothalamic injury. All (100% [30/30]) interview participants reported increased hunger after hypothalamic injury, with 97% (29/30) indicating that they or their child was less likely to feel full or never felt full (Table 2 and Box 1). Most interview participants (93% [28/30]) described intense, unrelenting hunger that led to increased food consumption and decreased control when eating. Caregivers further described the relentless nature of their child’s hunger and hyperfocus on food and associated food-seeking behaviors as the most bothersome aspects of acquired HO. Symptoms and impacts of acquired HO were described as contributing to social, familial, and mental/emotional burden (Box 1). Both trial participants and caregivers reported a constant struggle between the participant’s food-seeking behaviors and caregiver attempts to monitor and limit food, which led to family tension and stress. Symptoms of hyperphagia, such as unrelenting hunger and hyperfocus on food, were reported as some of the most bothersome symptoms associated with hypothalamic injury, in addition to the impacts of hyperphagia on social/family life (e.g., avoidance of uncontrolled food environment, negative emotional toll when missing out on activities due to hyperphagia) being reported as bothersome (Box 1).

**TABLE 2:** Impacts following hypothalamic injury reported by ≥ 25% of interview participants.

Impacts	Adult trial participants (*n* = 10)	Adolescent trial participants (*n* = 4)	Caregiver participants of trial participants aged < 12 years (*n* = 8)	Caregiver participants of trial participants aged ≥ 12 years (*n* = 8)	Total (*N* = 30)
Changes in weight					
Weight gain	100% (10/10)	100% (4/4)	100% (8/8)	100% (8/8)	100% (30/30)
Changes in hunger					
Change in hunger frequency					
More frequent	100% (10/10)	100% (4/4)	100% (8/8)	100% (8/8)	100% (30/30)
Change in hunger intensity					
More intense	90% (9/10)	75% (3/4)	100% (8/8)	100% (8/8)	93% (28/30)
No change or could not remember	10% (1/10)	25% (1/4)	0% (0/8)	0% (0/8)	7% (2/30)
Changes in eating habits					
Change in feeling full or satisfied					
Never felt full	30% (3/10)	50% (2/4)	75% (6/8)	50% (4/8)	50% (15/30)
Less likely to feel full	70% (7/10)	50% (2/4)	25% (2/8)	38% (3/8)	47% (14/30)
No change	0% (0/10)	0% (0/4)	0% (0/8)	13% (1/8)	3% (1/30)
Changes in control of eating					
Decreased control	90% (9/10)	75% (3/4)	100% (8/8)	100% (8/8)	93% (28/30)
No change	10% (1/10)	25% (1/4)	0% (0/8)	0% (0/8)	7% (2/30)
Change in amount of food eaten					
Increased amount	90% (9/10)	75% (3/4)	100% (8/8)	100% (8/8)	93% (28/30)
Changes in energy level					
Decreased energy level	90% (9/10)	100% (4/4)	100% (8/8)	100% (8/8)	97% (29/30)
Fatigue^a^	90% (9/10)	100% (4/4)	—	—	43% (13/30)
No change	10% (1/10)	0% (0/4)	0% (0/8)	0% (0/8)	3% (1/30)
Changes in physical activity					
Decreased physical activity	80% (8/10)	100% (4/4)	100% (8/8)	100% (8/8)	93% (28/30)
No change	20% (2/10)	0% (0/4)	0% (0/8)	0% (0/8)	7% (2/30)

*^a^*The concept of fatigue was probed only with patient participants (i.e., not with caregivers).

**Box 1:** Patient- and caregiver-reported experiences of hunger, eating habits, and effect of hyperphagia before treatment with setmelanotide in the TRANSCEND clinical trial.

Effect of weight gain and contributing factors	Experience with weight loss medications	Eating habits	Feelings of hunger/effect of hyperphagia on patients	Effect of hunger/hyperphagia on caregivers
“*I gained so much weight.… it’s been traumatizing for me. It was so scary. My weight went up almost 300 pounds and it’s been really hard.*” (Adolescent participant)	“*I lost some, but it didn’t work consistently for that long, so eventually we took it off.*” (Adult participant)	“*Every day. Food sneaking. My mom would find me climbing the gate we have. I’m walking off the front and [around] the back of the house just to get into the kitchen.*” (Adolescent trial participant)	“*At that time, it was just, when I was hungry, I was hungry, and I couldn’t really focus or think about anything else. I was very focused, very concentrated on I need food and I need it right now, and nothing else really mattered. At that point when I was stealing and buying, stealing, hiding food because of this constant, oh my God, if I don’t have food, I’m going to die, in a sense. It’s not necessarily what I was thinking, but it was a constant hunger.*” (Adult trial participant)	“*What bothered me the most is that she would be nearly inconsolable and obsessive about food. And any attempts to dissuade her from eating would result in her mental health spiraling and saying that she has the worst life and she wishes that the tumor had killed her. And she wishes that she was never born. And it has been the hardest part of all of the secondary effects of the tumor and the treatment to manage because it has led her to these uncontrollable emotional outbursts and led her to suicide ideation.*” (Caregiver participant; patient < 12 years)
“*Somehow it made social settings…very awkward for any kind of events outside of the home.…it’s not natural for people to be thinking about preventing someone from getting access to food. But everywhere we went we have to stop and scan the room to see what’s out, what’s in the trash cans, what’s in the drawers, what’s available for her. And is it a safe place? And so deciding…where she’s safe and how safe she is became a very difficult thing.…It impacted her relationship with her brother. It impacted her ability to have friendships.*” (Caregiver, participant ≥ 12 years)	“*He did [lose weight] in the beginning, but then it just kind of stopped. [Interviewer: Was it a significant amount, do you remember?] No, I don’t think so. I think he lost a little and then he was able to pretty much maintain the same weight.*” (Caregiver, adolescent participant)	“*She would eat until she was sick. So we had to really control how much she had access to food.*” (Caregiver participant; patient ≥ 12 years)	“*Oh, it was constant. It was nonstop. She was waking up in the middle of the night in her sleep crying out for food and saying she was hungry.*” (Caregiver participant; patient < 12 years)	“*What bothered me the most was how lonely I feel it was reflected on him…There was a tremendous sense of anxiety…for all of us to feel like every cupcake at a birthday party or every treat was making him sick…the worst is that you cannot enjoy food because it was making him sick…so that’s the hardest piece to reconcile, I think…Is that how sad and lonely food made all of us…*” (Caregiver participant; patient ≥ 12 years)
“*I used to play softball and basketball and a lot of different sports, but afterward, I wasn’t able to keep up with everyone else in those kinds of activities.…I still enjoyed them. It…felt like a disappointment, kind of, that I couldn’t do what I used to be able to do normally.*” (Adult participant)	“*The [GLP-1 receptor agonist], she started, and again, we saw really no results. So we actually decided to stop it probably after maybe 6 months.*” (Caregiver, adolescent participant)	“*…he would put anything in his mouth, Play-Doh, he’d go through the trash, he would go try to go through the fridge, he’d try to get into everything, it was just constant battle.*” (Caregiver participant; patient < 12 years)	“*One of the things that’s kind of embarrassing is that I was always so hungry, but on the other hand, I feel like I had to sneak it. I didn’t feel I could just eat openly. I had to sneak the food, hide it, eat when no one was looking.*” (Adolescent trial participant)	“The most, just not being able to have the freedom that we used to and seeing her be tortured by the hunger…Being able to go to the grocery store and just put your stuff in the cupboard or just not have to think about it so much and not worry about the next meal or plan things out. Just live the day, and when you’re hungry, you eat, and when you’re not, you don’t.” (Caregiver participant; patient ≥ 12 years)
“*…he didn’t have enough energy anymore to do anything. Before his brain tumor, he was so active, he had run around, he was a normal little boy, run around playing with the balls, always wanting to…we’d play like tee-ball and get in the pool and go swimming. Or we would go walking around the neighborhood and he would want to explore and do things. And afterward it’s like he didn’t feel like it, he didn’t feel like even playing sometimes, because he just didn’t have energy or just didn’t feel right.*” (Caregiver, participant < 12 years)			“*….the fridge and the cabinets were locked, and there were times when even it was locked and I would try to, in the middle of the night, I’d wake up and try to shove my arm inside the corner, the little crack of the cabinet and try to pull it open or try to sneak box of granola bars through the crack of the cabinet if I was able to pull the cable, loosen it up a little bit or whatever, that kind of stuff.…At nighttime, I’d say, ‘Hey, Mom, I’m locking the cabinet,’ and I’d show her I’d put the lock there, but I wouldn’t actually click the lock closed, so it looked like it was locked, but it wasn’t. And in the middle of the night, I woke up and I planned that I would go in there and see what food I could still get.…I’m not proud of it, but I used to try to steal food and money to get food because I was desperate and I felt like I needed it.*” (Adult trial participant)	“*… family members, especially in my family, eating is a big thing. And everybody wants to try and give you food. And here you have this child that has so much excitement about food. And so my family became an unsafe environment, like writ large. Just being around my family, period, was not safe for her.…It impacted her relationship with her brother. It impacted her ability to have friendships.*” (Caregiver participant; patient ≥ 12 years)
“*My energy level was super high before my surgery. I played tennis a lot and my mom always called me an Energizer Bunny. And after surgery, it dropped completely. I had no energy at all….I wanted to go back on the tennis court. I wanted to do all sorts of things. I wanted to do my craft, but I just had no energy to do anything.*” (Adult participant)			“*I was always hungry. My mom…had to put a door in our kitchen and lock it every day and night.…I would sneak food, hide it places.…I would sneak in the kitchen at nighttime and sneak food, hide food and eat food, food, food, food, food.…Food was my life. Food was everything.*” (Adolescent participant)	“*The hyperphagia is the worst thing of all. It is worse than the weight gain because it just takes over your life and literally it’s like an obsession. They can’t think, he wouldn’t even do his therapies or anything because he was just so wanting food, I guess.*” (Caregiver, participant < 12 years)
“*She would sleep all the time.…she takes several naps during the day. Really wouldn’t have a lot of energy to even go outside, which was something she previously loved to do. We would go to the pool, and she would last maybe half an hour whereas before she would be able to stay all day and play. So that was hard to watch too.*” (Caregiver, participant < 12 years)				“*Just the way it controlled her life. She was very, very heartbreaking to watch….She wouldn’t enjoy things. It was always in the back of her mind. Just completely control of her life. She’s not able to do things that other kids her age or other teenagers would do. She couldn’t get a job when she got a little older because she would steal food.…The hold that it had on her life was her most heartbreaking thing to see.*” (Caregiver, participant ≥ 12 years)
“*And then physical activity, [he] didn’t do very much, just didn’t want to play outside like he used to, didn’t want to go on his bike really like he used to or the scooter or anything like that. Kind of would just want to sit on the couch and watch TV…Definitely a big decrease in…how physically active he was.*” (Caregiver, participant < 12 years)				

#### 3.2.2 Factors contributing to weight gain

Additional factors reported as contributing to weight gain after hypothalamic injury included decreased energy level (97% [29/30]) and reduced physical activity (93% [28/30]; Box 1). Overall, both patients and caregivers reported experiencing substantial burden on their lives due to the changes in weight, hunger, energy level, and physical activity following hypothalamic injury.

#### 3.2.3 Medication use for weight loss following hypothalamic injury and before trial participation

Trial participants using weight loss medications before entering the trial were allowed to continue the treatment if the regimen had been stable for ≥ 3 months before the study, the dose was kept stable through the study, and if the participant had not experienced weight loss (> 2% for those ≥ 18 years or > 2% BMI for those < 18 years) in the previous 3 months. When asked about any previous experiences with medications to facilitate weight loss, 60% of participants (18/30) reported that they or their child had taken ≥ 1 such medication before trial enrollment, with the most commonly reported medications being stimulants (30% [9/30]) and GLP-1 receptor agonists (23% [7/30]). Of participants reporting prior weight loss medication use, only 28% (5/18) reported weight loss with those medications, which was described as limited and temporary (Box 1).

### 3.3 Participant- and caregiver-reported changes during the TRANSCEND trial

**FIGURE 1:**
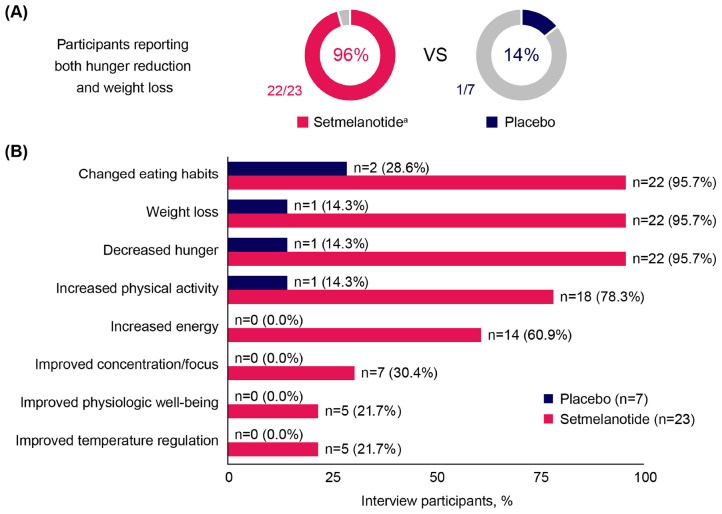
Change in hunger and weight with treatment initiation. **(A)** Interview participants reporting both decreased hunger and weight at Week 52. **(B)** Improvements reported during the trial by ≥ 2 interview participants. ^a^This number includes 1 caregiver participant of an individual aged < 12 years (on setmelanotide) who reported that the child maintained body weight over the course of the clinical trial but also grew taller.

After 52 weeks of treatment in the TRANSCEND trial, participants receiving setmelanotide reported broad improvements in hunger and weight. Participants in the setmelanotide group reporting reduced hunger and weight also consistently reported increased energy level and increased physical activity. Consistently, these improvements occurred at greater rates in the setmelanotide group than the placebo group (Figure 1 and Table 3). Among interview participants reporting improvements, the changes were reported to be extremely meaningful to the participants and their caregivers.

**TABLE 3:** Positive changes during the TRANSCEND trial reported by ≥ 2 participants^a^.

Changes	Adult trial participants (*n* = 10)		Adolescent trial participants (*n* = 4)		Caregiver participants of trial participants aged < 12 years (*n* = 8)		Caregiver participants of trial participants aged ≥ 12 years (*n* = 8)		Total (*N* = 30)	
	SET (*n* = 8)	PBO (*n* = 2)	SET (*n* = 4)	PBO (*n* = 0)	SET (*n* = 6)	PBO (*n* = 2)	SET (*n* = 5)	PBO (*n* = 3)	SET (*n* = 23)	PBO (*n* = 7)
Weight loss^a^	8	0	4	0	6	0	4	1	96% (22/23)	14% (1/7)
Changed eating habits	8	0	4	0	6	1	4	1	96% (22/23)	29% (2/7)
Increased feeling full or satisfied	8	0	4	0	6	1	4	0	96% (22/23)	14% (1/7)
Increased control of eating	8	0	2	0	6	0	4	1	87% (20/23)	14% (1/7)
Decreased amount of food eaten	7	0	4	0	4	1	4	0	83% (19/23)	14% (1/7)
Decreased hunger	8	0	4	0	6	0	4	1	96% (22/23)	14% (1/7)
Decreased hunger frequency	8	0	4	0	6	0	4	0	96% (22/23)	0% (0/7)
Decreased hunger intensity	8	0	4	0	5	0	2	1	83% (19/23)	14% (1/7)
Increased physical activity	6	0	4	0	5	0	3	1	78% (18/23)	14% (1/7)
Increased energy	3	0	3	0	5	0	3	0	61% (14/23)	0% (0/7)
Decreased fatigue^b^	3	0	3	0	–	–	–	–	26% (6/23)	0% (0/7)
Improved concentration/focus	–	–	1	0	4	0	2	0	30% (7/23)	0% (0/7)
Improved physiologic wellbeing	1	0	–	–	3	0	1	0	22% (5/23)	0% (0/7)
Improved temperature regulation	1	0	1	0	2	0	1	0	22% (5/23)	0% (0/7)

PBO, placebo; SET, setmelanotide.

*^a^*Includes interview participants who reported positive changes after responding “yes” to “are there any changes in eating habits” during interviews. Includes 1 caregiver of a trial participant < 12 years in the setmelanotide group who reported their child maintained weight over the course of the trial but also grew taller.

*^b^*Thirteen trial participants (9 adults and 4 adolescents) reported experiencing fatigue after hypothalamic injury.

#### 3.3.1 Impacts on weight

Overall, 77% (23/30) of interview participants reported weight loss during the trial. Among trial participants self-reporting weight loss in the interviews (*n* = 12; all in the setmelanotide group), adult participants (*n* = 8) lost an average of 21.8 (range, 3.2–38.6) kg, and adolescent participants (*n* = 4) lost an average of 16.8 (range, 9.1–34.0) kg. Caregivers of patients in the setmelanotide group aged < 12 years (*n* = 6) reported that their child lost an average of 6.4 (range, 3.6–11.3) kg, and caregivers of patients in the setmelanotide group aged ≥ 12 years (*n* = 4) reported that their child lost an average of 18.6 (range, 2.7–45.4) kg. One caregiver of a trial participant in the placebo group (≥ 12 years) reported that their child lost 4.5 kg.

#### 3.3.2 Impacts on hunger/hyperphagia

**FIGURE 2:**
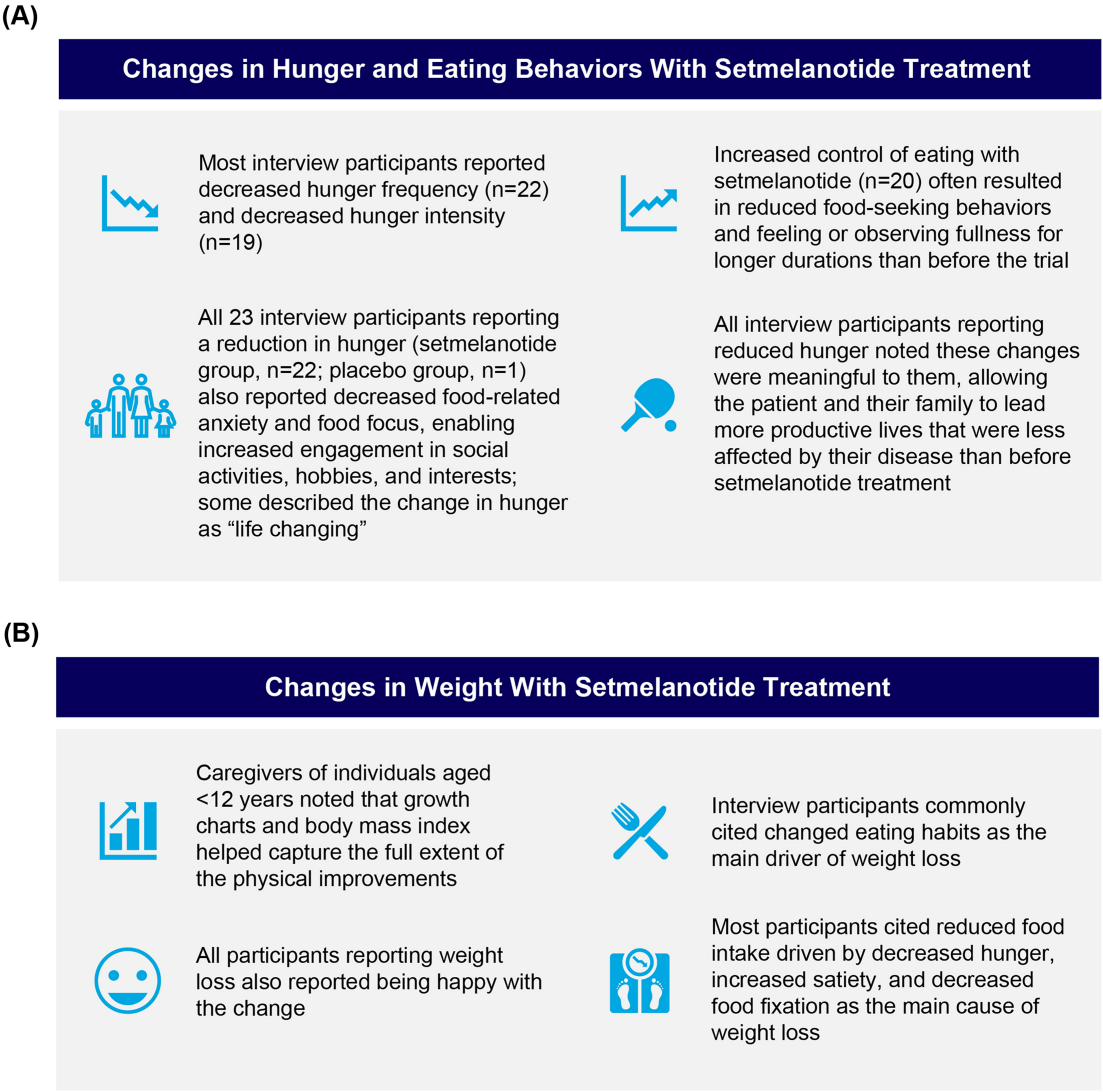
Trends regarding changes in **(A)** hunger and **(B)** weight following setmelanotide treatment.

Most participants receiving setmelanotide reported improvements in both hunger and weight (96% [22/23]) compared with 14% [1/7] of participants receiving placebo (Figure 1). All participants reporting reduced weight were content with the weight change (100% [23/23]), citing changed eating habits as the main driver of weight loss (Figure 2). Among participants who experienced or observed reductions in hunger, patient participants described hunger that occurred less often and, when present, felt less intense; caregiver participants reported that their child asked for food far less often than before starting the clinical trial and could be redirected more easily (Box 2). Parents also observed improvements in their child’s food-seeking behaviors, including fewer attempts to sneak or steal food and greater emotional stability when food was not allowed or available (Box 2). Reductions in hunger also positively affected self-esteem, social and family relationships, and focus.

**Box 2:** Patient- and caregiver-reported experiences of hunger, eating habits, and effect of hyperphagia after treatment with setmelanotide in the TRANSCEND clinical trial.

Changes in weight and eating habits	Feelings of hunger/effect of hyperphagia on patients	Effect of hunger/hyperphagia on caregivers	Activity and energy level	Additional changes reported
“*Compared to before, I don’t snack as much during the day or anything like that. I’ll just eat my meals and usually be good.*” (Adolescent trial participant; setmelanotide)	“*[My hunger is] definitely less intense. Before the study, if I felt hungry, it was almost like I had to eat. I almost felt like, ‘Oh my gosh, I’m going to die if I don’t eat. I’m going to starve to death if I don’t eat.’ Now I don’t feel that way. If I feel hungry, if I have access to food, we eat. If I have to wait, then it’s okay.*” (Adult trial participant; setmelanotide)	“*It helped our relationship. We don’t argue about food that much anymore or amounts or what she’s going to have if we go eat out. It was always very stressful about picking something good to eat, healthier to eat. It was always arguments or the amount.*” (Caregiver participant, patient ≥ 12 years; setmelanotide)	“*Imagine going from having to sleep all day because you have no energy to do anything. When you are awake, you’re constantly worried about what when your next meal is going to be there, even if you have a snack right next to you to taking that all away. What kid wants to sit there and talk about food and what they’re going to eat. That’s all that her brain would allow her to focus on. To now when she’s able to participate in things and not have that constant worry. And she has that energy back. Again, I don’t know how else to describe it besides life-changing for her. Absolutely changed her life.*” (Caregiver, participant < 12 years; setmelanotide)	“*It’s crazy how much this medicine has helped me, and it’s normalized all my blood work.*” (Adult participant; setmelanotide)
“*I feel like she has complete control now.…So we don’t worry about it anymore….We don’t worry about what the healthy options are because we know that she can control herself and won’t even be drawn to those things.*” (Caregiver participant, patient < 12 years; setmelanotide)	“*She would say, ‘I’m not hungry,’ which never came out of her mouth before. My gosh. The first time she said it, I was like, ‘What’d you just say?*”’ (Caregiver participant, patient ≥ 12 years; setmelanotide)	“*I think that I get a little more freedom because she is a little more independent. Not having to watch her so much is a big relief.*” (Caregiver participant, patient ≥ 12 years; setmelanotide)	“*I run up stairs. I don’t walk up stairs. I help around the house. I’m able to do chores. I’m able to work outside with my mom, with the garden, and help my dad. We were just washing my car outside in the hot sun. I’m able to walk around the neighborhood, walk the dog. It’s the exact opposite.*” (Adult participant; setmelanotide)	“*I’m able to be in class and learn and pay attention, ask questions. I’m able to have conversations about real life things instead of food being my life.*” (Adolescent participant; setmelanotide)
“*Sometimes she will say, ‘Oh, I don’t want to eat this. I’m going to save this for later. I’m full.’ She acts normal. Like I’ve said, ‘[name deleted], I’m hungry. Are you ready for dinner?’ She goes, ‘I’m a little bit hungry, but not too bad.’ Something like that. I think it’s been life-changing for her.*” (Caregiver participant, patient ≥ 12 years; setmelanotide)	“*I think that he would say he feels full. And he knows when he is full. He can’t finish food. Like if we order takeout from a restaurant and he orders a bowl, he can never finish the bowl, whereas before he would finish the entire bowl.*” (Caregiver participant, patient ≥ 12 years; setmelanotide)	“*The impact it’s had on us as a whole family as well.…But that’s also been just such a huge change for our family and a lot happier, healthier balance overall. One less stressor for the whole family that used to be quite a source of tension.…Prior to her diagnosis….We loved to travel, we loved to be outdoors. When everything happened, we couldn’t do those things anymore. It was hard to travel because of all of her medical complexities and the hunger. And she just wouldn’t also have the energy or the interest.…And since starting the trial, I feel like we can do things and enjoy things as a family. We’ve gone on several vacations.…We’re back to being outdoors.*” (Caregiver participant, patient < 12 years; setmelanotide)	“*Again, it’s hard to put into words. We went from a summer of sleeping through everything to a summer of her participating in everything. So just life-changing for her, for us, for our whole family.*” (Caregiver, participant < 12 years; setmelanotide)	“*It’s also huge for me as a parent and our entire family unit because it was really hard, even on the sibling.…But the stark contrast between them, it got to be really, really hard where it’s like ‘How come [name deleted] gets to eat rice and I don’t?’…[Now] we can go out to dinner together.…We can go and have meals at other people’s houses….So I think it’s changed a lot for our family dynamic and our abilities to do more things.*” (Caregiver, participant < 12 years; setmelanotide)
“*She stopped pretty much all of the food-seeking behaviors we were seeing.…She was more redirectable away from the food, which she never was before. So that was really good.*” (Caregiver, participant < 12 years; setmelanotide)	“*He’s not asking for food. When he gets done eating, he’ll actually say, ‘I’m full,’ and he won’t finish meals oftentimes. And when he wakes up in the morning, he’s not in here asking for food first thing. So that’s definitely changed, how much he asks for food throughout the day has decreased. And then, like I said, he’ll stop when he’s full now, so that’s better.…But not only has his eating improved, and his hunger has become more normalized, and he’s not seeking out food as much. When he gets hungry around snack time, he’ll come into the kitchen and look for something, but he doesn’t do it like he did before. Before, he would go and he’d be trying to open the fridge. He’d be trying to open the pantry. He’d be trying to get in the trash. He’d be trying to get anything. And now that has improved.*” (Caregiver, participant < 12 years; setmelanotide)	“*…we have unlocked everything and she’s around food more than she was before but chooses not to eat it.…we could have a birthday party and she doesn’t want the cake. There’s access, but she doesn’t want it.*” (Caregiver, participant ≥ 12 years; setmelanotide)	“*His energy has definitely improved. Where before I told you all he’d want to do at home was kind of sit in his chair and watch TV, and now he’s moving all day long.*” (Caregiver, < 12; setmelanotide)	“*Yeah, I think [the improvements were] across the board. That preoccupation was constantly focused on, was food focus before. So now that she doesn’t have that preoccupying her brain, I feel like she can attend to whatever she wants to attend to. So whether that be schoolwork, chores at home, activities that she wants to do.*” (Caregiver, participant < 12 years; setmelanotide)
“*[Now] she’ll tell me she’s hungry in the morning and for lunch, and before we started the trial, she was telling me she was hungry every hour at the very least. Sometimes several times in the same half an hour. Sometimes every 5 min. So you’re going from 20 to 30 times a day or more to 2 or 3. That’s a huge change.*” (Caregiver, < 12 years; setmelanotide)	“*It’s life-changing because now he started to be more like himself again, and he’s participating in his therapies.…when we started the trial he was in a wheelchair, and now he’s able to walk by himself and did a lot of that I’m sure is because his energy has been better, and he’s been able to participate in his therapies, and he started to talk again. And I know that’s because he is not distracted, just looking for the next meal. He’s able to focus and do everything that he’s supposed to do.…And when we’re at home he wants to play.…He wants to do his normal little things, and he’s not just thinking about food anymore.*” (Caregiver, participant < 12 years; setmelanotide)	“*All these changes have been positive. Him not focused on where the next meal is going to be is what allows him to have more of a normal life. And us too, because we’re not having to argue with him all the time, we don’t have to tell him, ‘No, you can’t eat right now. No, we’re not going to eat right now.*”’ (Caregiver, participant < 12 years; setmelanotide)	“*Now, I’m actually able to go on the playground….I’m able to be involved with other good activities, it’s a beautiful thing in my life, I am able to be involved.*” (Adolescent participant; setmelanotide)	“*Now that she is able to be more social again…they call her ‘the mayor’ at school. Because everyone knows her; she knows everyone. Like she says hi to everyone. She’ll stop and chat with anyone. It’s really funny, and it’s really awesome and it’s great to see her in her element, doing her thing. Socially, that’s been great for her.…In many ways, it’s changed for the better.*” (Caregiver, participant < 12 years; setmelanotide)
“*I feel totally in control….[It’s] very rare that I have to go back for seconds. If I just eat a proper serving, I feel satisfied and move on.*” (Adult participant; setmelanotide)	“*Oh man, this change was like, I don’t even know how to say it. It’s like huge, beyond. I remember being so miserable and just horrible about myself, if you will. And so now I feel good.*” (Adolescent participant; setmelanotide)	“*The fact that she’s a normal kid thanks to 1 shot a day. I can’t properly communicate how profound that is, the fact that it was a 2-year nightmare, but it’s essentially over as long as we have these meds. That’s wild. It’s why I was focusing on hunger earlier, because you can’t properly appreciate the after until you understand the before. And the fact that there is a before and after and it’s that clear is thanks to… this medicine.*” (Caregiver, participant < 12 years; setmelanotide)	“*I’m able to get more done and I just feel more productive when I’m able to do things that I enjoy doing outside of my work.*” (Adult participant; setmelanotide)	
“*She’s tall for her age.…she’s height-weight appropriate now….none of the kids she’s meeting this year as new kindergartners know what her medical history is because, visually, she is indistinguishable from every other 5- and 6-year-old.*” (Caregiver, < 12 years; setmelanotide)	“*She’s not constantly clamoring and stressing over food and when her next meal will be and what she can eat and how much she can eat. She’s not in distress constantly.*” (Caregiver, < 12 years; setmelanotide)		“*He can play a round of golf, can play 9 holes of golf, and then shoot baskets in the driveway, and then still stay up to like 11:00 pm.*” (Caregiver, ≥ 12 years; setmelanotide)	
“*Before, I was so heavy, [it was] hard for me to walk so I needed a wheeling backpack, but now I’m able to use an actual backpack.*” (Adolescent participant; setmelanotide)	“*[My changes in hunger] are so meaningful because I can talk to people about real-life, everyday situations. [Before, all conversation focused on] food, food, food.*” (Adolescent participant; setmelanotide)		“*Being able to go outside and just have energy to do things during the day, being able to be normal again. It’s awesome.*” (Adult participant, setmelanotide)	
“*My weight is now at 140, and I started at like 225. It’s crazy how much this medicine has helped me.*” (Adult participant; setmelanotide)	“*I totally lost my hunger. Went down like crazy. I was just, I was skipping meals. [Laughter.] It was a very strange thing for my whole family…I was losing a lot of weight. I wasn’t that interested in food.*” (Adult participant; setmelanotide)		“*She’s definitely stronger too.…she is independent with stairs now.…Getting in and out of the car is no longer a problem at all.…she’s a kid who should be able to do those things. And it’s so nice that she finally can.*” (Caregiver, < 12 years; setmelanotide)	
“*She is glad that she’s losing weight. It seems like at least in that regard, she’s enjoying these moments with friends and family.*” (Caregiver, ≥ 12 years; setmelanotide)			“*It’s meaningful to me because instead of staying inside 24/7 in bed, I get to go outside and walk with my grandpa or my mom.*” (Adolescent participant; setmelanotide)	
			“*She’s no longer taking any naps whatsoever during the day. She stays wide awake.*” (Caregiver, < 12 years; setmelanotide)	

#### 3.3.3 Changes in activity and energy level

**FIGURE 3:**
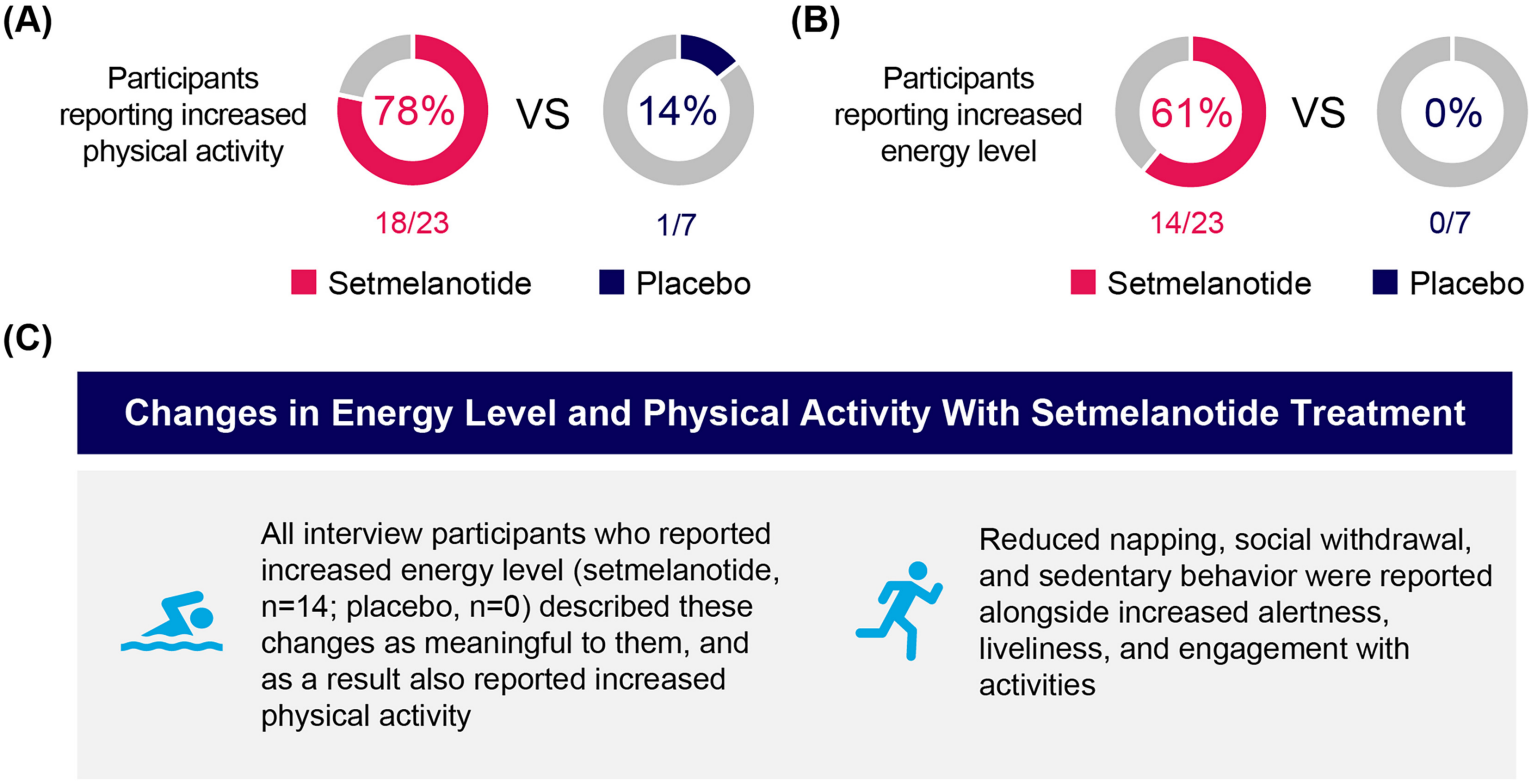
Change in energy level with treatment initiation. **(A,B)** Interview participants reporting increased physical activity and energy level at Week 52. **(C)** Insights and overall trends of energy level changes with setmelanotide treatment.

In the setmelanotide group, a greater proportion of participants reported increased physical activity (78% [18/23]) and energy level (61% [14/23]) than those in the placebo group (14% [1/7] and 0% [0/7], respectively; Figure 3). All participants who reported increased energy level described these changes as meaningful to them due to better quality of life and increased physical activity. Participants described feeling less tired, being more alert, and being better engaged in activities such as work or school (Figure 3C). Caregivers of patients < 12 years old observed that their child no longer needed to take naps during the day, showed more interest in hobbies and sports, and generally displayed more “pep” (Box 2).

#### 3.3.4 Additional changes reported: trial participants

Improved concentration/focus and improved physiologic wellbeing were reported by 30% (7/23) and 22% (5/23) participants receiving setmelanotide, respectively, whereas no placebo-treated participants reported these improvements. Spontaneous reports of other improvements from interview participants in the setmelanotide group included improved ability to engage in hobbies, social activities, and play (13.0% [3/23]) and improvements in confidence, self-esteem, and independence (22% [5/23]). Clinical improvements such as better blood laboratory results or reduction in medications used were also spontaneously reported (22% [5/23]; Box 2).

#### 3.3.5 Additional changes reported: caregivers

Additional changes during the trial reported by caregivers included broad improvements across their child’s social life, work/school, or ability to concentrate. Among the 11 caregivers who reported any improvement in their child’s social life, school or work life, or ability to concentrate, 91% (10/11) reported improved social life (9 in the setmelanotide group), 64% (7/11) reported improved work/school performance (6 in the setmelanotide group), and 54% (6/11) reported increased ability to concentrate (6 in the setmelanotide group). Furthermore, caregiver burden was reduced with setmelanotide treatment, with caregivers stating they experienced reduced stress and anxiety, increased work performance, and improved focus on personal health (Box 2).

#### 3.3.6 Perceived meaningfulness and impact of the changes

All interview participants reporting reduced hunger and weight noted the changes they experienced were meaningful (100% [23/23]), with some participants describing them as “life changing,” and stated that these improvements allowed the trial participant and their family to lead life less impacted by acquired HO (Box 2).

## 4 Discussion

Acquired HO confers substantial negative burden on both patients and caregivers because of reduced energy level, accelerated and sustained weight gain, and hyperphagia (Abuzzahab et al., 2025; Argente et al., 2025; Kayadjanian et al., 2023; Roth and McCormack, 2024). Results from this TRANSCEND clinical trial substudy show treatment with setmelanotide provided more extensive benefits beyond hunger and weight management in acquired HO compared with those who received placebo. Participants who received setmelanotide reported increased physical activity, higher energy level, and improvements in emotional wellbeing, social interactions, work/school performance, and focus; lesser or no substantial benefits were reported in these areas for those receiving placebo. These changes were consistent across adolescent and adult participants. Furthermore, participants found these changes meaningful in allowing them and their families to experience less disease-related impact on daily life.

These data provide a unique lens for viewing the patient experience and disease burden associated with acquired HO. Following hypothalamic injury, patients and their caregivers consistently described substantial weight gain, disruptive food-seeking behaviors, hunger, decreased energy level and physical activity, and negative impacts on overall QOL. While hyperphagia has been associated with acquired HO, (Heymsfield et al., 2025) the subjective nature of hunger can be difficult to fully capture with clinical measures. These qualitative interviews shed light on the severity and impact of hyperphagia symptoms in acquired HO. Further, because this study explores a population who previously would have had “normal” hunger and energy level before the injury, the reported impairments highlight the severe burden of disease compared with preinjury levels.

The interview results were consistent with and highly supportive of the clinical data, with participants who received setmelanotide consistently reporting substantial improvements in weight, hunger, and energy level compared with those receiving placebo. In the primary analysis of the TRANSCEND trial, both adult and pediatric patients receiving setmelanotide also had significant improvements in Impact of Weight on Quality of Life Questionnaires (Lite-Clinical Trial version and Kids version), as well as the Symptoms of Hyperphagia composite score after approximately 52-week of treatment (Miller et al., 2026). Moreover, the safety profile of setmelanotide was consistent with that previously reported in a Phase 2 trial of HO and trials of other conditions (Clément et al., 2020; Collet et al., 2017; Haqq et al., 2022; Roth et al., 2024a). The substudy interview data presented here support and expand on these findings, suggesting benefits beyond weight and hunger reduction for individuals with acquired HO. Further, the patient- and caregiver-reported benefits associated with setmelanotide in patients with acquired HO mirror those reported by patients with genetic forms of rare MC4R pathway–associated obesity (Ervin et al., 2023; Wabitsch et al., 2022). These data provide support for the salience of key outcomes (i.e., hunger/hyperphagia, reduced energy levels, QOL) across these populations. Further, these data highlight the importance of exploring the patient and caregiver perspective via qualitative studies to identify and confirm what constitutes a treatment benefit and perceived meaningful improvement for their conditions.

Limitations of this substudy include potential recall bias and proxy reporter bias, which is a potential limitation of similarly designed interview-based research. The sample size (*N* = 30) reflects the number of participants expected to achieve saturation of key themes in the qualitative interview. Participants who elected to enroll in the interview portion of the study may have been more motivated than the overall study population (*n* = 120) because they may have noticed changes attributable to the active drug. However, few participants declined to participate in the substudy; thus, the first 30 who consented generally reflected randomization. Additionally, because only participants from US-based study sites were included, the findings may not be broadly generalizable to other global regions. Despite these limitations, the qualitative results of this study support the substantial burden of acquired HO and the efficacy of setmelanotide with respect to patient health and function.

These findings are highly supportive of the Phase 3 TRANSCEND trial efficacy outcomes seen with setmelanotide. Specifically, these qualitative data support the importance of hunger, weight, energy level, and QOL improvements in this patient population. While receiving setmelanotide treatment, most interview participants reported substantial changes that they perceived as impactful across each of these key areas and described marked improvements in overall QOL. These interviews both confirm the relevance of the clinical efficacy outcomes as well as highlight then need for efficacious treatments for acquired HO.

## Data Availability

The datasets presented in this article are not readily available because the raw data supporting the conclusions of this article will be made available by the authors, upon reasonable request. Requests to access the datasets should be directed to Jieruo Liu, jliu@rhythmtx.com.

## Footnotes

1
https://atlasti.com/
