# Supplementary material for: Quality of life improvements with setmelanotide treatment in acquired hypothalamic obesity: TRANSCEND trial interview results from US participants
Source: Front Behav Neurosci. 2026 Jul 15;20:1830070. doi: 10.3389/fnbeh.2026.1830070 (PMC13415934; doi:10.3389/fnbeh.2026.1830070)
Supplement: Supplementary file 1 [file Data_Sheet_1.pdf]

RM-493-040 In-Trial Interviews in Hypothalamic Obesity

Patient Interview Guide

RTI-HS Project No. 0307219

Preface

Note that this is a semistructured interview guide, not a script. The questions included in this guide will frame the discussion and ensure that specific topics are addressed in a standardized manner; however other questions and follow-up probes may also be asked as the goal is to maintain a conversational approach.

Introduction

[Introduce self and notetaker then briefly reiterate the purpose and format of the interview.]

First, thank you again for speaking with us today. During the interview, we will ask about your hunger, energy, and other weight-related experiences before and after your hypothalamus was damaged. We will then talk about any changes you've experienced since you started the Phase 3 HO clinical trial (which we will call the "main study").

We'll be doing similar interviews with up to 30 other individuals who participated in the main study to help us better understand the impacts of hypothalamus damage and how, if at all, these impacts changed during the main study. As a reminder, your participation is completely voluntary, and you may end the discussion at any time. We expect today's interview to last about 75 minutes. Please feel free to ask us any questions you may have or request a break in the interview at any time. While we have a series of topics we wish to cover with you, we also intend for these interviews to be conversational.

With your permission, we will audio record today's interview to make sure we do not miss any important information and to help us create a transcript of the interview. All information you provide to us will be kept confidential. While the transcripts will be provided to the study sponsor, all names and any other identifying information will first be removed from these documents.

Please keep in mind that there are no wrong answers to our questions. We appreciate the opportunity to learn from your experiences and are truly grateful for your time.

Before we begin, do you have any questions?

Okay, I am going to go ahead and start recording.

START RECORDING

I am now audio recording. Do I have your permission to continue with the audio-recorded interview?

- ☐ Yes → Continue
- ☐ No → **STOP INTERVIEW**

Experiences Before the Clinical Trial

First, we would like to talk about your experiences after the damage to your hypothalamus, but before starting treatment with the study medication about 14 months ago.

1. About how old were you when the damage to your hypothalamus occurred?
2. Did your weight change after this damage occurred?
   - [If yes] As best you can remember, about how much weight did you lose or gain since [the time of damage to hypothalamus]?

Changes in Hunger and Eating Behaviors

3. How, if at all, did your hunger change after the damage to your hypothalamus (but before you started the clinical trial about 14 months ago)?
   - Did your hunger feel more or less intense?
   - Did you feel hungry more or less often during the day?
4. Were you more or less likely to feel full or satisfied after eating?
5. How would you describe your ability to control how much and what you ate <u>after</u> the damage to your hypothalamus (but before starting the clinical trial)?
   - Did you have more or less control over your eating?
6. How, if at all, did your ability to control how much and what you ate change or vary from day to day?
7. How if at all, did how much or what you ate change after the damage to your hypothalamus. Please describe.

Changes in Energy and Physical Activity

8. Did you notice a change in your energy level after the damage to your hypothalamus (but before starting the clinical trial)? Please describe what you noticed about your energy level.

   - [If less energy reported; but not the word "fatigue"] Did you experience fatigue after the damage and before starting the clinical trial?

     – [If yes] In your own words, what does "fatigue" mean to you? How, if at all, is fatigue related to energy?

9. Was there a change in your physical activity after the damage to your hypothalamus?
   [If needed] By physical activity, we mean any kind of activity that involves some physical exertion including daily activities like walking to the mailbox, doing household chores, or taking the stairs, as well as more formal physical activities like exercise, walking/running, and playing sports.

   - [If yes] What changed? Why?
   - Did you find you were doing more or less physical activity? Please describe the changes.

Treatment Aspirations

10. Why did you choose to participate in the clinical study? What changes were you hoping to see?

Experiences During the Clinical Trial

Next, we would like to hear about your experiences while taking the study medication (and how you feel today). When we are talking about your experiences while taking the study medication, we are talking about your experiences during the past 14 months or so, since [MONTH].

11. Has your weight changed since you started the clinical trial?
    [Note: probe for change in body mass index (BMI) if weight loss not reported]

    - [If yes] Approximately how much weight have you lost or gained?
    - Why do you think you [lost or gained] weight? What was different, if anything, about how you felt?

Changes in Hunger and Eating Behaviors

12. How has your hunger changed, if at all, since you started the clinical trial?
    - Does your hunger feel more or less intense?
    - Do you feel hungry more or less often during the day?
    - Are you more or less likely to feel full or satisfied after eating?

13. How would you describe your ability to control how much and what you eat now? Please tell us more about that.
    - [If change in hunger] How meaningful are the changes you have experienced in your hunger since starting the study medication? Why?

14. Since starting the clinical trial, have you changed what and/or how much you eat? Please tell me what has changed, if anything, and why?

Changes in Energy and Physical Activity

15. How, if at all, has your energy level changed since starting the clinical trial?
    - [If fatigue endorsed before taking the clinical trial] How, if at all, has your fatigue changed?

16. How, if at all, has your level of physical activity changed since you started the clinical trial?
    [If needed] Remember, physical activity can include many daily activities such as walking to the mailbox, doing household chores, or taking the stairs, as well as more formal exercise such as playing sports, walking, and running.
    - Are you more or less physically active now? In what ways?

17. [If changes experienced in energy or physical activity – ask the following question for each change] How meaningful are the changes you have experienced in your [energy and/or physical activity] since starting the clinical trial? Why?

Cognitive Debriefing

Now I'd like to turn our attention to a few questions that you answered during the clinical trial. I'm going to share the first question on my screen and, when I do, I'd like you to please read it out loud and then think out loud as you come up with your answer. I'd really like to learn what's going through your mind as you think about what the question

is asking and how you choose your answer. I'll also have a few follow-up questions as we go along.

Hunger Items

In the last 24 hours, on average, how hungry did you feel?

0	1	2	3	4	5	6	7	8	9	10
Not hungry at all										Hungriest possible

18. In your own words, what is this question asking?

19. Please tell me why you selected [response]. What does that mean to you?

20. Thinking back to before you started the clinical trial but after the damage to your hypothalamus (about 14 months ago), how do you think you would have answered this question on a typical day?

    - *[If change]* Please tell me why you might have answered [response] before the clinical trial. What does [response] mean to you?
        - How would you describe the change from [participant's response from start of the study] to [current response]? How meaningful, if at all, is this change? Why?

In the last 24 hours, how hungry did you feel when you were the most hungry?

0	1	2	3	4	5	6	7	8	9	10
Not hungry at all										Hungriest possible

21. In your own words, what is this question asking? How, if at all, is this different from the previous question?

22. To understand your experiences with hunger, do you think it is important to ask about your average level of hunger, as well as your maximum level of hunger? Why or why not?

23. Please tell me why you selected [response]. What does that mean to you?

24. Thinking back to before you started the clinical trial but after the damage to your hypothalamus (about 14 months ago), how do you think you would have answered this question on a typical day?

    - *[If change]* Please tell me why you might have answered [response] before the clinical trial. What does [response] mean to you?

– How would you describe the change from [participant's response from start of the study] to [current response]? How meaningful, if at all, is this change? Why?

Global Hunger Question: Hunger Severity

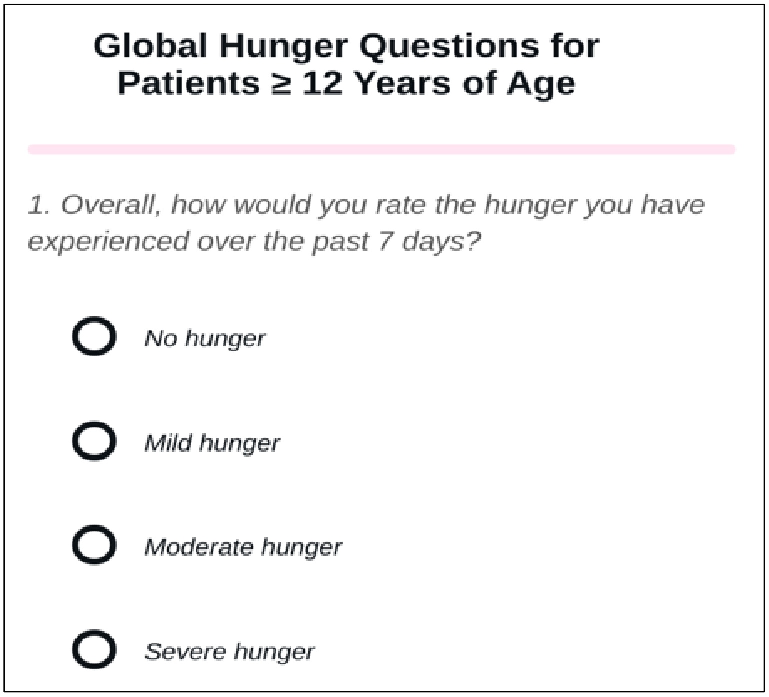
Global Hunger Questions for Patients ≥ 12 Years of Age

1. Overall, how would you rate the hunger you have experienced over the past 7 days?

- *No hunger*
- *Mild hunger*
- *Moderate hunger*
- *Severe hunger*

25. In your own words, what is this question asking?

26. Please tell me why you selected [response]. What does that mean to you? [If "No hunger" is selected, follow up (as needed) to understand if this is seen as a treatment benefit or not. If not clear from initial responses, further explore meaning of "No hunger" – for example, does this mean you did not experience hunger for 7 days; you did not experience extreme hunger for 7 days; or something different.]

27. Thinking back to before you started the clinical trial but after the damage to your hypothalamus (about 14 months ago), how do you think you would have answered this question at the end of a typical week?

    - *[If change]* Please tell me why you might have answered [response] before the clinical trial. What does [response] mean to you?
        – How would you describe the change from [participant's response from start of the study] to [current response]? How meaningful, if at all, is this change? Why?

   - *[If change is not meaningful]* What amount of change would be meaningful to you? Why?

- *[If no change OR change ≠ 1-category improvement]* If you had changed from [participant's response] to [1-category improvement], how would you describe this change? Would this be a meaningful change to you? Why or why not?
   - *[If 1-category improvement not meaningful]* How would you describe a change from [participant's response] to [2-category improvement]? Would this be a meaningful change to you? Why or why not?

Global Hunger Question: Hyperphagia Severity

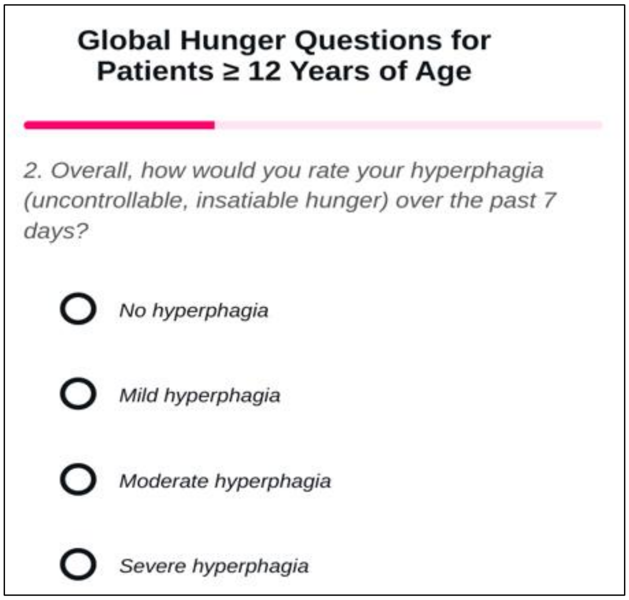

Global Hunger Questions for Patients ≥ 12 Years of Age

2. Overall, how would you rate your hyperphagia (uncontrollable, insatiable hunger) over the past 7 days?

- *No hyperphagia*
- *Mild hyperphagia*
- *Moderate hyperphagia*
- *Severe hyperphagia*

28. In your own words, what is this question asking? What does "hyperphagia" mean to you? Were you familiar with this term before starting the clinical trial?

29. What, if anything, is the difference to you between hunger and hyperphagia?

30. Please tell me why you selected [response]. What does that mean to you?

31. Thinking back to before you started the clinical trial but after the damage to your hypothalamus, how do you think you would have answered the question at the end of a typical week?
    - *[If change]* Please tell me why you might have answered [response] before the clinical trial. What does [response] mean to you?

- How would you describe the change from [participant's response from start of the study] to [current response]? How meaningful, if at all, is this change? Why?
- *If change is not meaningful]* What amount of change on this scale would be meaningful to you? Why?

- *[If no change OR change ≠ 1-category improvement]* If you had changed from [participant's response] to [1-category improvement], how would you describe this change? Would this be a meaningful change to you? Why or why not?
  - *[If 1-category improvement not meaningful]* How would you describe a change from [participant's response] to [2-category improvement]? Would this be a meaningful change to you? Why or why not?

Global Hunger Question: Hunger Change

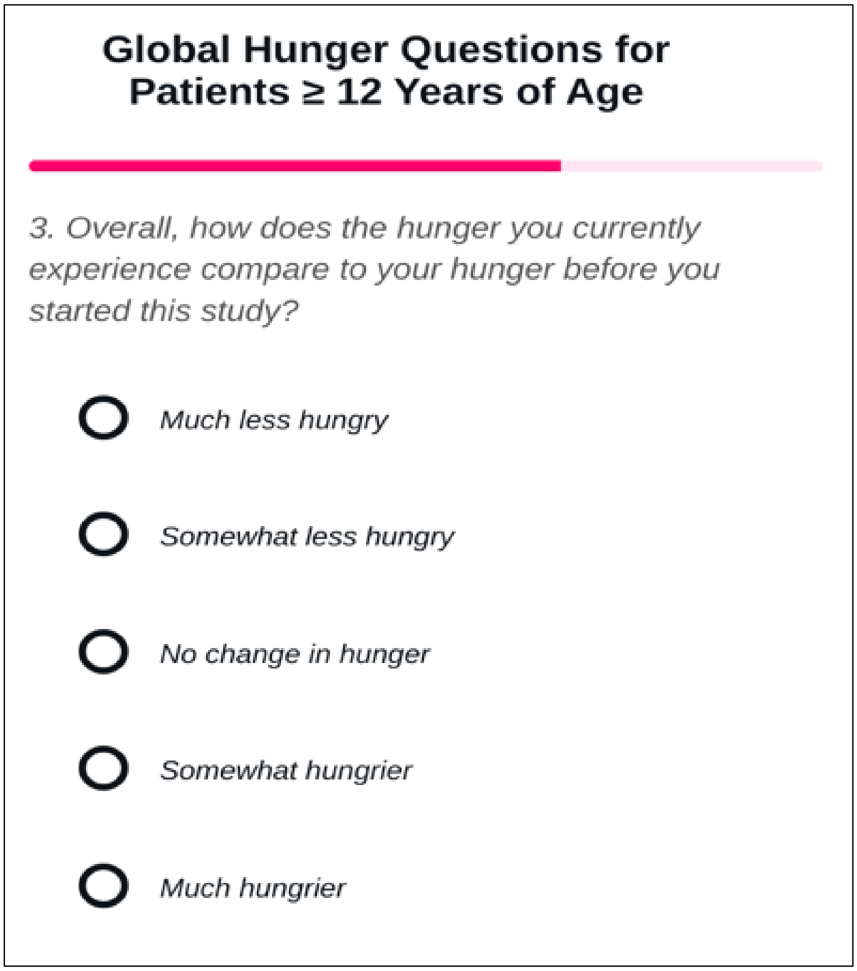

Global Hunger Questions for Patients ≥ 12 Years of Age

3. Overall, how does the hunger you currently experience compare to your hunger before you started this study?

- ○ *Much less hungry*
- ○ *Somewhat less hungry*
- ○ *No change in hunger*
- ○ *Somewhat hungrier*
- ○ *Much hungrier*

32. In your own words, what is this question asking?

33. Please tell me why you chose [response]. What does [response] mean to you?

- *[If change]* How meaningful, if at all, is this change? Why?
- *[If answer ≠ Somewhat less hungry]* If you had answered "Somewhat less hungry," would that correspond to a meaningful improvement? Why or why not?

Use of Weight Loss Medications

Before we let you go, we have just a few quick questions.

34. Did you take any prescription medication for weight loss after your hypothalamus was damaged (and before starting the clinical trial)?

- *[If yes]* How many prescription medications did you take for weight loss after the damage to your hypothalamus?
- [For each medication]
  - What was the name of the medication?
  - How long did you take the medication?
  - How much weight did you lose while taking the medication?

Closing

Is there anything else about your experiences or any feedback related to the studies you have been participating in that you would like to share with us?

The sponsor of the study asked us to thank you, on their behalf, for sharing your valuable thoughts and experiences with us. Thank you so much!
