# Supplementary material for: Quality of life improvements with setmelanotide treatment in acquired hypothalamic obesity: TRANSCEND trial interview results from US participants
Source: Front Behav Neurosci. 2026 Jul 15;20:1830070. doi: 10.3389/fnbeh.2026.1830070 (PMC13415934; doi:10.3389/fnbeh.2026.1830070)
Supplement: Supplementary file 2 [file Data_Sheet_2.pdf]

RM-493-040 In-Trial Interviews in Hypothalamic Obesity

Caregiver Interview Guide

RTI-HS Project No. 0307219

Preface

Note that this is a semistructured interview guide, not a script. The questions included in this guide will frame the discussion and ensure that specific topics are addressed in a standardized manner; however other questions and follow-up probes may also be asked as the goal is to maintain a conversational approach.

Introduction

[Introduce self and notetaker then briefly reiterate the purpose and format of the interview.]

First, thank you again for speaking with us today. During the interview, we will ask you to share what you have observed in relation to your child's hunger, energy, and other weight-related experiences before and after their hypothalamus was damaged. We will then talk about any changes you've observed since your child started the Phase 3 HO clinical trial (which we will call the "main study").

We'll be doing similar interviews with up to 30 other individuals (including patients and caregivers) who participated in the main study to help us better understand the impacts of hypothalamus damage and how, if at all, these impacts changed during the main study. As a reminder, your participation is completely voluntary, and you may end the discussion at any time. We expect today's interview to last about 75 minutes. Please feel free to ask us any questions you may have or request a break in the interview at any time. While we have a series of topics we wish to cover with you, we also intend for these interviews to be conversational.

With your permission, we will audio record today's interview to make sure we do not miss any important information and to help us create a transcript of the interview. All information you provide to us will be kept confidential. While the transcripts will be provided to the study sponsor, all names and any other identifying information will first be removed from these documents.

Please keep in mind that there are no wrong answers to our questions. We appreciate the opportunity to learn from your experiences and are truly grateful for your time.

Before we begin, do you have any questions?

Okay, I am going to go ahead and start recording.

START RECORDING

I am now audio recording. Do I have your permission to continue with the audio-recorded interview?

- ☐ Yes → Continue
- ☐ No → **STOP INTERVIEW**

Experiences Before the Clinical Trial

First, we would like to talk about your child's experiences after the damage to their hypothalamus, but before starting treatment with the study medication about 14 months ago.

1. About how old was your child when the damage to their hypothalamus occurred?

2. Did your child's weight change after this damage occurred?
   - [If yes] As best you can remember, about how much weight did they lose or gain since [the time of damage to hypothalamus]?

Changes in Hunger and Eating Behaviors

3. How, if at all, did your child's hunger seem to change after the damage to their hypothalamus (but before they started the clinical trial about 14 months ago)?
   - Did their hunger seem to be more or less intense? How could you tell?
   - Did they seem to be hungry more or less often during the day? How did you know?

4. Did they ever seem to feel full or satisfied after eating? How could you tell?

5. To what extent did your child seem able to control how much and what they ate after the damage to their hypothalamus (but before starting the clinical trial)?
   - Did they seem to have more or less control over their eating? How could you tell?

6. How, if at all, did their ability to control how much and what they ate seem to change or vary from day to day?

7. How, if at all, did how much or what they ate change after the damage to their hypothalamus. Please describe.

8. What, if anything, bothered you the most about the change in your child's hunger after the damage to their hypothalamus? Why?

Changes in Energy and Physical Activity

9. Did you notice a change in your child's energy level after the damage to their hypothalamus (but before starting the clinical trial)? Please describe what you observed about your child's energy level.

10. Was there a change in your child's physical activity after the damage to their hypothalamus?
    [If needed] By physical activity, we mean any kind of activity that involves some physical exertion including daily activities like walking to the mailbox, doing household chores, or taking the stairs, as well as more formal physical activities like exercise, walking/running, and playing sports.
    - [If yes] What changed? What did you notice in your child's activity level after the damage to their hypothalamus but before the clinical trial?
    - Did you observe your child doing more or less physical activity? Please describe the changes.

Treatment Aspirations

11. Why did you decide to allow your child to participate in the clinical study? What changes were you hoping to see?

Experiences During the Clinical Trial

Next, we would like to hear about your child's experiences while taking the study medication (and how they are currently doing). When we are talking about your child's experiences while taking the study medication, we are talking about their experiences during the past 14 months or so, since [MONTH].

12. Has your child's weight changed since they started the clinical trial?
    [Note: probe for change in body mass index (BMI) if weight loss not reported]
    - [If yes] Approximately how much weight have they lost or gained?

- Why do you think your child [lost or gained] weight? What changes, if any, in their behavior did you notice?

Changes in Hunger and Eating Behaviors

13. How, if at all, has your child's hunger changed since they started the clinical trial? [If changes noticed]
    - Do they seem to feel more or less hungry during the day? Please tell me more about that.
    - Do they seem to feel hungry more or less of the time? Please tell me more about that.
    - How do you think your child feels now after eating a meal?
      – Do they ever seem to feel full or satisfied after eating? What do you notice?
      – How, if at all, has the frequency in which they seem to be full after eating changed since starting the clinical trial? Please describe.
14. To what extent does your child seem to be able to control how much and what they eat now? Please tell us more about that.
    - [If change in hunger] How meaningful are the changes you have observed in your child's hunger since starting the study medication? Why?
15. Since starting the clinical trial, have you observed changes in what and/or how much your child eats? Please tell me what has changed.

Changes in Energy and Physical Activity

16. How, if at all, has your child's energy level changed since starting the clinical trial? What have you noticed?
17. How, if at all, has your child's level of physical activity changed since they started the clinical trial (about 14 months ago)?
    [If needed] Remember, physical activity can include many daily activities such as walking to the mailbox, doing household chores, or taking the stairs, as well as more formal exercise such as playing sports, walking, and running.
    - Is your child more or less physically active now? In what ways, what have you noticed?

18. [If changes experienced in energy or physical activity – ask the following question for each change] How meaningful are the changes you have observed in your child's [energy and/or physical activity] since starting the clinical trial? Why?

Treatment Impacts

19. What (other) benefits, if any, has your child experienced since taking participating in the clinical trial?

    [If not mentioned spontaneously, probe on the following aspects:]

    - Social (relationships, participation)
    - Work/school (participation, productivity)
    - Concentration (ability to focus, sit still, complete tasks)

20. [If any benefits reported] How, if at all, have these changes impacted you? How, if at all, have these changes impacted your family/family dynamics and/or relationship?

    - [If improvements] What do you think is the most important benefit your child experienced while participating in the clinical trial?
    - [If child experienced improvements] What, if any, is the most important benefit/impact you experienced while your child participated in the clinical trial?

Cognitive Debriefing

Now I'd like to turn our attention to a few questions that you may have answered during the clinical trial. I'm going to share the first question on my screen and, when I do, I'd like you to please read it out loud and then think out loud as you come up with your answer. I'd really like to learn what's going through your mind as you think about what the question is asking and how you choose your answer. I'll also have a few follow-up questions as we go along.

Caregiver Reported Global Hunger Question: Hunger Severity

1. *How hungry has your child behaved in the past 7 days?*
☐ *Not hungry at all*
☐ *A little hungry*
☐ *Moderately hungry*
☐ *Extremely hungry*

21. In your own words, what is this question asking?

22. Please tell me why you selected [response]. What does that mean to you? [If "Not hungry at all" is selected, follow up (as needed) to see if this is seen as a treatment benefit or not. If not clear from initial responses, further explore meaning of "No hunger" – for example, does this mean the child never showed desire for food; a lack of behaviors associated with extreme hunger; or something different.]

23. Thinking back to before your child started the clinical trial but after the damage to their hypothalamus (about 14 months ago), how do you think you would have answered this question at the end of a typical week?
    - *[If change]* Please tell me why you might have answered [response] before the clinical trial. What does [response] mean to you?
        - How would you describe the change from [participant's response from start of the study] to [current response]? How meaningful, if at all, is this change? Why?
        - *[If change is not meaningful]* What amount of change would be meaningful? Why?
    - *[If no change OR change ≠ 1-category improvement]* If your child had changed from [participant's response] to [1-category improvement], how would you describe this change? Would this be a meaningful change? Why or why not?
        - *[If 1-category improvement not meaningful]* How would you describe a change from [participant's response] to [2-category improvement]? Would this be a meaningful change? Why or why not?

Caregiver Reported Global Hunger Question: Hunger Change

2. How hungry is your child currently behaving compared to before starting this study?
- ☐ *Much less hungry*
- ☐ *Somewhat less hungry*
- ☐ *No change in hunger-related behavior*
- ☐ *Somewhat more hungry*
- ☐ *Much more hungry*

24. In your own words, what is this question asking?

25. Please tell me why you chose [response]. What does [response] mean to you?
    - *[If change]* How meaningful, if at all, is this change? Why?
    - *[If answer ≠ Somewhat less hungry]* If you had answered "Somewhat less hungry," would that correspond to a meaningful improvement? Why or why not?

Use of Weight Loss Medications

Before we let you go, we have just a few quick questions.

26. Did your child take any prescription medication for weight loss after his/her hypothalamus was damaged (and before starting the clinical trial)?
    - *[If yes]* How many prescription medications did your child take for weight loss after the damage to his/her hypothalamus?
    - [For each medication]
        - What was the name of the medication?
        - How long did your child take the medication?
        - How much weight did your child lose while taking the medication?

Closing

Is there anything else about your experiences or any feedback related to the study your child has been participating in that you would like to share with us?

The sponsor of the study asked us to thank you, on their behalf, for sharing your valuable thoughts and experiences with us. Thank you so much!
